# Genetic Variation of the Ghrelin Signalling System in Individuals with Amphetamine Dependence

**DOI:** 10.1371/journal.pone.0061242

**Published:** 2013-04-08

**Authors:** Petra Suchankova, Elisabet Jerlhag, Nitya Jayaram-Lindström, Staffan Nilsson, Kjell Toren, Annika Rosengren, Jörgen A. Engel, Johan Franck

**Affiliations:** 1 Department of Pharmacology, The Sahlgrenska Academy at University of Gothenburg, Gothenburg, Sweden; 2 Department of Clinical Neuroscience, Division of Psychiatry at Karolinska Institutet, Stockholm, Sweden; 3 Department of Mathematical Statistics, Institute of Mathematical Sciences at Chalmers University of Technology, Gothenburg, Sweden; 4 Department of Occupational and Environmental Medicine, Sahlgrenska Academy at University of Gothenburg, Gothenburg, Sweden; 5 Department of Molecular and Clinical Medicine, Sahlgrenska Academy at University of Gothenburg, Gothenburg, Sweden; Yale University, United States of America

## Abstract

The development of amphetamine dependence largely depends on the effects of amphetamine in the brain reward systems. Ghrelin, an orexigenic peptide, activates the reward systems and is required for reward induced by alcohol, nicotine, cocaine and amphetamine in mice. Human genetic studies have shown that polymorphisms in the pre-proghrelin (*GHRL*) as well as GHS-R1A (*GHSR*) genes are associated with high alcohol consumption, increased weight and smoking in males. Since the heritability factor underlying drug dependence is shared between different drugs of abuse, we here examine the association between single nucleotide polymorphisms (SNPs) and haplotypes in the *GHRL* and *GHSR*, and amphetamine dependence. *GHRL* and *GHSR* SNPs were genotyped in Swedish amphetamine dependent individuals (n = 104) and controls from the general population (n = 310). A case-control analysis was performed and SNPs and haplotypes were additionally tested for association against Addiction Severity Interview (ASI) composite score of drug use. The minor G-allele of the *GHSR* SNP rs2948694, was more common among amphetamine dependent individuals when compared to controls (*p_c_* = 0.02). A significant association between the *GHRL* SNP rs4684677 and ASI composite score of drug use was also reported (*p_c_* = 0.03). The haplotype analysis did not add to the information given by the individual polymorphisms. Although genetic variability of the ghrelin signalling system is not a diagnostic marker for amphetamine dependence and problem severity of drug use, the present results strengthen the notion that ghrelin and its receptor may be involved in the development of addictive behaviours and may thus serve as suitable targets for new treatments of such disorders.

## Introduction

Amphetamine and its derivatives are widely abused drugs and amphetamine dependence is of great concern in today's society. The development of drug dependence, such as for amphetamine, largely depends on the effects of amphetamine in the brain reward systems including the mesolimbic dopamine system (for review see [1]). These systems are important in mediating the rewarding properties of natural incentives, e.g. food, as well as of drugs, such as alcohol and amphetamine [2]–[4]. The drug response to amphetamine is heterogeneous and depends on factors such as expectancies of the drug, psychiatric disorders or personality traits, gender differences as well as genetics (for review see [5]). The heritability factor underlying drug dependence is shared between different drugs of abuse [6]–[8], and we hypothesize that ghrelin and its receptor, growth hormone secretagogue receptor (GHS-R1A), may be common denominators.

Ghrelin, a circulating orexigenic stomach-derived hormone, regulates energy homeostasis mainly via hypothalamic GHS-R1A [9]–[13]. Centrally or peripherally administered ghrelin has also been shown to activate the reward systems, specifically the cholinergic-dopaminergic reward link [14]–[18], and may thereby increase the incentive value of motivated behaviours such as food seeking. Supportively, ghrelin signalling has been implicated in hedonic feeding as well as in food-motivated behaviours [19]–[24].

In addition, central ghrelin signalling is required for the reinforcing properties of drugs of abuse as supported by preclinical findings (for review see [25] or [26]). Specifically, a GHS-R1A antagonist attenuates the rewarding properties of alcohol, nicotine, cocaine and amphetamine as measured by locomotor stimulation, accumbal dopamine release and condition place preference [27]–[29]. In rodents, ghrelin has been shown to enhance cocaine-induced locomotor stimulation, to condition a place preference for cocaine and to induce cocaine-seeking behaviours [30]–[32]. Moreover, ghrelin increases, and a GHS-R1A antagonist decreases the consumption and operant self-administration of alcohol in rodents [28], [33], [34].

We have obtained support for these preclinical data in human genetic studies in which single nucleotide polymorphisms (SNPs) and haplotypes in the pre-proghrelin (*GHRL*) as well as GHS-R1A (*GHSR*) genes were associated with high alcohol consumption, increased weight and smoking in males as well as with personality traits related to alcohol use disorder in males with an alcohol use disorder [35]–[37]. Additionally, associations between a *GHRL* haplotype and paternal heredity of alcohol use disorder in females as well as increased weight in alcohol dependent males has been reported [37]. Haplotypes of the ghrelin signalling system have also been linked to increased sucrose consumption [23]. Given the shared genetic contribution to drug abuse, that polymorphisms in ghrelin related genes are associated with alcohol intake and smoking and that GHS-R1A antagonists blocks amphetamine-induced reward in mice, we hypothesise that genetic variations in the *GHRL* and *GHSR* may be associated with amphetamine dependence in humans. Thus, in the present association study SNPs and haplotypes of the *GHRL* and *GHSR* were investigated in Swedish individuals with a Diagnostic and Statistical Manual of Mental Disorders, 4^th^ edition, (DSM-IV) diagnosis of amphetamine dependence and who had additionally been assessed using the Addiction Severity Index (ASI).

## Methods

### Subjects

All individuals with a history of amphetamine dependence (26 women, 78 men) were recruited at the Stockholm Centre for Dependency Disorders (Beroendecentrum Stockholm), Sweden. The individuals had a DSM-IV diagnosis of amphetamine dependence and reported amphetamine as their primary drug of choice. They did not have a current diagnosis of any other major psychiatric disorders (such as bipolar, schizophrenia) or major somatic disorder (e.g. heart disease). Subjects testing positive for any other illicit substance were excluded. Data on nicotine use was not systematically collected on all patients, however, a majority (85%) were dependent on nicotine. The mean age of the patients was 40.3 years (SD 10.5, range 20–60). All patients gave their written informed consent to the study and the protocol was approved by the regional ethics review board at Karolinska Institutet, Stockholm, Sweden.

The control subjects consist of individuals (214 women, 96 men) randomly selected among a larger sample of low alcohol consuming individuals (i.e. self-reported alcohol consumption of 0.3–2.3 g alcohol/day) recruited for the INTERGENE study (see [36] for detailed description of this selection). These controls are Caucasians and did not self-report any drug use. However, 54 (out of 308 i.e. 17.8%) reported that they were current smokers. INTERGENE is a population based research program with the main objective of assessing the INTERplay between GENEtic susceptibility and environmental factors in the risk of developing chronic diseases in western Sweden [38]–[40]. The mean age of the selected control subjects was 56.6 years (SD 13.5, range 20–76). All control subjects gave their written informed consent to the study and the protocol was approved by the regional ethics review board at University of Gothenburg, Gothenburg, Sweden.

### Clinical Assessment

The individuals in the present study are Caucasians and fulfilled the diagnosis of amphetamine dependence based on the DSM-IV criteria. In addition to the DSM-IV interview, patient alcohol and drug usage patterns were measured via the Addiction Severity Interview (ASI), a structured interview which maps the severity and duration of dependence across seven sub-scales [41]. 27 subjects were not interviewed with the ASI. All individuals included had the DSM-IV diagnosis of current amphetamine dependence. They did not have co-diagnosis of any other substance dependence (Table 1). 51 of 62 individuals met ASI criteria for previous heavy alcohol consumption (more than 4 drinks for women and 5 for men, per occasion) in the past year. The urine toxicology of the patients confirmed that all patients were current D/L-amphetamine users. The average length of use was 13 years of regular usage (>4 days per week). Consumption ranged between 1–2 grams per occasion (average consumption of 6 grams per week). These amounts are typical for chronic amphetamine dependent individuals treated at the clinic. See table 1 for data on patient substance abuse characteristics.

**Table 1:** Patient Substance Abuse Characteristics.

n = 62–77*		Mean (SD)
Age of onset amphetamine abuse		20.1 (7.5)
ASI Composite score for drug use^1^		0.17 (0.1)

	Usage^2^ (%)	Mean (SD)^3^
Amphetamine	100	15.3 (10.3)
Intravenous usage^4^	76.0	15.6 (11.7)
Any alcohol	75.8	13.4 (11.6)
Heroin	16.6	4.8 (4.7)
Pain relievers (opiates or equivalent)	24.3	4.3 (4.0)
Sedatives	29.7	6.4 (6.1)
Cocaine	25.0	2.9 (2.1)
Cannabis	62.0	10.2 (8.3)
Hallucinogenics	21.4	3.5 (4.5)
Inhalants	10.4	3.1 (4.8)
Multiple drug use	57.6	12.1 (10.4)

ASI, addiction severity index.

* Variation in n due to variation in number of individuals responding to the ASI items.

1 The composite score ranges from 0 (no problems) to 1 (severe problems).

2 Percentage users out of valid responses.

3 Data expressed as mean (SD) of number of years in life that the drug has been abused (subjects who had not taken the given drug were excluded).

4 The ASI does not specify which drug was used intravenously.

### SNP selection and genotyping

The Tag SNP selection procedure has been described in detail previously [37]. Briefly, genotyping data for the pro-ghrelin and GHS-R1A genes for the Caucasian CEPH population was downloaded from the International Haplotype Mapping Project web site (http://www.hapmap.org). Six Tag SNPs in the pro-ghrelin gene (rs4684677, rs42451, rs35680, rs3491141, rs696217, and rs26802) and four Tag SNPs in the GHS-R1A gene (rs2948694, rs572169, rs2232165, and rs495225) were selected by using the Tagger function in the Haploview software with the r^2^ set to a minimum of 0.80 (for their location and the SNPs which they tag). Out of these SNPs rs4684677 rs3491141, rs696217, rs572169, and rs495225 were force-included due to previous studies (for refs, see [37]).

The genotyping procedure has been described in detail previously [37]. Human genomic DNA was extracted from blood samples from both patients and controls using the QIAamp 96 DNA Blood Blood Kit (Qiagen, Hilden, Germany). The studied SNPs were genotyped from the obtained genomic DNA using TaqMan Pre-Designed SNP Genotyping Assays® (Applied Biosystems, Foster City, CA) on the ABI PRISM 7900HT Sequence Detection System (Applied Biosystems) using the TaqMan Allelic Discrimination technology [42]. In the TaqMan Pre-Designed SNP Genotyping Assays® a polymerase chain reaction amplifies the region around the SNPs. This polymerase chain reaction also includes two oligonucleotide probes, specific for one SNP allele each. These probes are labelled with one fluorescent reporter dye each (VIC and FAM) at one end and a quencher that absorbs fluorescence at the other end. When the DNA polymerase extends the newly synthesized DNA strand, it cleaves any probe that is tightly bound to the DNA, causing an increase in fluorescence. After polymerase chain reaction amplification the signal strength of each reporter molecule is measured and displayed in a scatter plot with the fluorescence of the probes are plotted on the X- and Y-axis. Given that each point represents one sample the genotype of each sample can be determined. For details on the studied Tag SNPs and the TaqMan assay ID, see table 2.

**Table 2:** Studied Single Nucleotide Polymorphisms.

Gene	SNP	Position^1^	Alleles	SNP location	SNP type	TaqMan SNP assay
*GHRL*	rs4684677	10328453	A/T	Exon 3	Missense (Gln90Leu)	C__25607748_10
	rs42451	10330377	G/A	Intron 2	Intron	C____965982_10
	rs35680	10330564	G/A	Intron 2	Intron	C___3151002_10
	rs696217	10331457	G/T	Exon 2	Missense (Leu72Met)	C___3151003_20
	rs34911341	10331548	T/C	Exon 2	Missense (Arg51Gln)	C__25607739_20
	rs26802	10332365	G/T	Promoter	Intron	C___3151004_10
*GHSR*	rs2948694	172165163	A/G	Intron 1	Intron	C__16174361_10
	rs572169	172165727	G/A	Exon 1	Silent mutation	C___1079489_20
	rs495225	172166033	T/C	Exon 1	Silent mutation	C___1079488_1_
	rs2232165	172166144	C/T	Exon 1	Silent mutation	C__15857645_10

*GHRL = *pre-proghrelin gene; *GHSR* = growth hormone secretagogue receptor gene; SNP = single nucleotide polymorphism.

1 Position on chromosome 3 for the studied SNPs in *GHRL* and *GHSR*.

### Statistical analyses

Deviation from Hardy-Weinberg equilibrium (HWE) was assessed for all genotyped SNPs in cases and controls separately. Allele frequencies were compared between amphetamine dependent patients and controls using Chi2-test. Homogeneity of odds ratios was tested with a Breslow-Day test. The possible influence of the studied SNPs on the ASI composite score of drug use was investigated using linear regression with sex and age as covariates.

For each gene, haplotype analyses were performed including all SNPs using stepwise logistic regression in the case-control analysis and linear regression including sex and age as covariates in the ASI composite score of drug use analysis. Haplotype frequencies were estimated using the expectation-maximisation algorithm [43]. A significance level of 0.05 was used. Due to LD between SNPs correction for multiple testing was carried out using 10 000 permutations in each gene (i.e. six in *GHRL* and four in *GHSR1*). Corrected *p*-values are designated as *p_c_*. The statistical analysis was carried out using SPSS for Mac (Version 19.0.0.1, SPSS, Chicago, IL, USA) and HelixTree (Version 6.3, Golden Helix, Bozeman, MT, USA).

## Results

All studied SNPs in both the patient and control group had a HWE p-value>0.01. As seen in table 3, the minor allele of the rs2948694 SNP located in intron 1 of the *GHSR* gene was associated with amphetamine dependence (*p_c_* = 0.02, allelic OR 1.84). When men and women were analysed separately for rs2948694 the ORs were 1.48 and 2.11 respectively, but not significantly different (*p* = 0.5). When the control group was split into non-smokers and current smokers the minor allele frequencies were 10.0% and 13.0% respectively. Pairwise comparisons of the three groups then gave: Amphetamine users vs smokers (OR = 1.47,p = 0.25), smokers vs non-smokers (OR = 1.33, p = 0.37) and amphetamine users vs non-smokers (OR = 1.96, p = 0.004).

**Table 3:** Distribution of minor allele frequencies (%) of the studied SNPs in controls and in amphetamine dependent individuals.

	Allele	Controls	Cases	OR	*p*-value^1^	*p_c_*-value
	minor (major)	*n* = 275-308*	*n* = 103**			
**GHRL SNPs**						
rs4684677	A (T)	9.3	9.2	0.99	0.98	1
rs42451	A (G)	24.2	28.6	1.26	0.21	0.70
rs35680	G (A)	45.6	43.7	0.92	0.63	1
rs696217	T (G)	10.9	8.3	0.73	0.28	0.84
rs34911341	T (C)	1.5	1.0	0.66	0.59	0.99
rs26802	G (T)	26.6	30.1	1.19	0.33	0.88
**GHSR SNPs**						
rs2948694	G (A)	10.6	18.0	1.84	**0.006**	**0.02**
rs572169	A (G)	35.0	31.6	0.86	0.37	0.81
rs495225	C (T)	26.6	27.7	1.05	0.77	0.99
rs2232165	T (C)	2.9	1.9	0.65	0.44	0.88

*GHRL = *pre-proghrelin gene; *GHSR* = growth hormone secretagogue receptor gene; SNPs = single nucleotide polymorphisms.

* Variation in *n* due to failed genotyping (n = 275 for rs42451 and rs35680 and n≥306 for the remaining SNPs). Total n for controls is 310.

** one sample failed genotyping.

1 Chi-square test of allele frequencies.

Single marker analysis also showed association between the minor allele (90Leu) of the *GHRL* SNP rs4684677 and increased ASI composite scores for drug use (table 4).

**Table 4:** Association between ASI composite score for drug use among amphetamine dependent individuals and GHRL and GHSR SNPs.

	β^1^ (*95% CI*)	*p*-value^2^	*p_c_-*value
**GHRL SNPs**			
rs4684677	0.07(0.02–0.12)	**0.007**	**0.03**
rs42451	−0.01(−0.04–0.03)	0.723	0.99
rs35680	−0.01(−0.04–0.02)	0.336	0.88
rs696217	0.10(−0.10–0.30)	0.344	0.89
rs34911341	0.02(−0.04–0.07)	0.512	0.98
rs26802	0.01(−0.04–0.03)	0.731	0.99
**GHSR SNPs**			
rs2948694	0.00(−0.04–0.04)	0.921	1
rs572169	0.01(−0.03–0.04)	0.719	0.99
rs495225	0.01(−0.03–0.04)	0.700	0.98
rs22232165	−0.03(−0.14–0.09)	0.655	0.98

CI = confidence interval; *GHRL = *pre-proghrelin gene; *GHSR* = growth hormone secretagogue receptor gene.

1 Change in ASI per minor allele.

2 Linear regression controlling for age and sex.

Although associations were found both between a haplotype in the *GHSR* gene and diagnosis as well as a haplotype in the *GHRL* gene and ASI composite scores for drug use these analyses did not contribute to additional information next to the single marker analysis.

## Discussion

The present study is, to our knowledge, the first investigating the *GHRL* and *GHSR* genes, encoding ghrelin and its receptor, in relation to amphetamine dependence and problem severity of drug use. We found that the minor allele of a SNP located in *GHSR* (rs2948694) is associated with amphetamine dependence. This finding was strengthened by the fact that the control smokers had an allele frequency intermediate between non-smokers and patients. We also found a significantly higher ASI drug composite score in patients carrying the minor allele of a *GHRL* SNP (rs4684677).

Common neurobiological mechanisms for the rewarding properties of both drugs of abuse and food have been suggested in both animal and human studies [4], [44], [45], and the ghrelin signalling system could be one of the mediators of this effect. This may be one reason for the high co-morbidity between eating disorders and drug dependence [46]. Both preclinical and human genetic findings have previously suggested an important role for GHS-R1A in drug-induced reward (*vide infra*). Specifically, the ability of alcohol, nicotine, cocaine as well as amphetamine to induce locomotor stimulation, increase accumbal dopamine release and to condition a place preference, are attenuated by GHS-R1A antagonists in mice [28], [29], [47]. Previous human genetic studies have reported association between SNPs and haplotypes of the *GHSR* gene and heavy alcohol consumption as well as body mass index in heavy alcohol consuming individuals [37]; smoking and type II alcohol dependence in women [36] as well as with self-directedness in alcohol dependent individuals [35]. Collectively, these data suggest that GHS-R1A may be involved in drug reward, drug intake as well as characteristic traits of addictive behaviours and the receptor may therefore be a potential target for treatment of various addictions. It has been suggested that either the constitutive activity of the GHS-R1A [48] or the ability of the GHS-R1A to form a heterodimer with dopamine D1-like or D2 receptors [49], [50] may affect the set point of the mesolimbic dopamine system. The functional role of the studied SNP is not known and the significance of these results cannot be determined. However, we hypothesize that the SNP in the GHS-R1A gene could, tentatively, influence the activity of tegmental dopamine neurons and thereby influence the ability of these dopamine neurons to be activated by addictive drugs. However, this needs to be further elucidated.

We also found that patients with the uncommon variant (90Leu) of the *GHRL* SNP (rs4684677) have higher ASI composite scores for drug use. Our previous human genetic studies have reported associations between SNPs and haplotypes of the *GHRL* gene and body mass index in heavy alcohol consuming individuals [37]; paternal alcohol dependence and withdrawal symptoms in female alcohol-dependent individuals [36] and with self-transcendence in alcohol dependent individuals [35], and ghrelin may thus be implicated in mechanisms underlying drug use. Preclinical data support an important role of ghrelin in drug intake and drug-reward. In mice, ghrelin administration into brain ventricles or locally into reward nodes (ventral tegmental area or laterodorsal tegmental area) increases the intake of alcohol [28] and alcohol-induced reward is attenuated in ghrelin knockout mice [47]. Moreover, the ability of cocaine to increase locomotor activity and condition a place preference is enhanced by ghrelin in mice and high plasma levels of ghrelin is associated with cocaine-seeking behaviours in rats [30]–[32]. Although hypothetical, it could be speculated that this SNP, if resulting in higher ghrelin levels, could lead to drug seeking or craving, as ghrelin plasma levels have been previously shown to be higher during alcohol abstinence [51] and craving for alcohol [52].

Interestingly ghrelin and its receptor appear to be important for reward in general. Specifically, suppression of the GHS-R1A reduces reward induced by, the intake of and motivation to consume palatable foods [21], [22]. It has also been shown that ghrelin and its receptor regulates the intake of and motivation to consume sucrose and saccharin [24], [53], [54], at the level of the mesolimbic dopamine system [55]. Human functional magnetic resonance imaging studies show that ghrelin administration increases the natural response to food in reward related areas, including the mesolimbic dopamine system [56]. In addition, human genetic findings show an association between a haplotype in the *GHRL* gene and high sucrose consumption [23]. Taken together with the data regarding ghrelin, the GHS-R1A and drugs of abuse it may be suggested that ghrelin and its receptor may have an important role in regulating reward in general and that GHS-R1A may be a potential target for new treatments of addictive behaviours such as compulsive overeating.

The current study is faced with limitations, the main being the small sample size, the possibility of a type 1 error and population stratifications. Therefore our study should be considered as a pilot study and additional studies replicating our data are warranted. Another confounding factor may be that the controls self-reported nicotine and alcohol usage via a questioner. Unreported usage of addictive drugs could therefore tentatively affect the obtained results. Additionally, the modest sample size may limit the power to detect effects. However, the consistency in our findings where a SNP in *GHSR* and *GHRL* were associated with amphetamine dependence and ASI drug composite score, may suggests an important role for ghrelin and its receptor in drug dependence. Given previous reports showing associations between the studied SNPs and heavy alcohol-use as well as smoking [37] and the comorbidity between amphetamine, alcohol and nicotine dependence, we cannot rule out the possibility of an interaction between the studied SNPs and alcohol and/or nicotine dependence. Additionally, the functional value of the studied SNPs is yet to be determined. Although our research is highly hypothesis driven, the findings reported here must be regarded as preliminary until replicated.

In conclusion, even though genetic variability of ghrelin and its receptor is not diagnostic marker for amphetamine dependence and problem severity of drug use, the present results still strengthen the notion that ghrelin and the GHS-R1A are involved in some of the mechanisms underlying the development of addictive behaviours.
